# Ambipolar blend-based organic electrochemical transistors and inverters

**DOI:** 10.1038/s41467-022-33264-2

**Published:** 2022-09-22

**Authors:** Eyal Stein, Oded Nahor, Mikhail Stolov, Viatcheslav Freger, Iuliana Maria Petruta, Iain McCulloch, Gitti L. Frey

**Affiliations:** 1 Department of Materials Science and Engineering, Technion – Israel Institute of Technology, Haifa, 32000 Israel; 2 The Wolfson Department of Chemical Engineering, Technion – Israel Institute of Technology, Haifa, 32000 Israel; 3 Department of Chemistry, Chemistry Research Laboratory, University of Oxford, Oxford, OX1 3TA UK; 4 Physical Sciences and Engineering Division, KAUST Solar Center (KSC), King Abdullah University of Science and Technology (KAUST), Thuwal, 23955-6900 Saudi Arabia

**Keywords:** Electronic devices, Organic molecules in materials science, Electronic properties and materials

## Abstract

CMOS-like circuits in bioelectronics translate biological to electronic signals using organic electrochemical transistors (OECTs) based on organic mixed ionic-electronic conductors (OMIECs). Ambipolar OECTs can reduce the complexity of circuit fabrication, and in bioelectronics have the major advantage of detecting both cations and anions in one device, which further expands the prospects for diagnosis and sensing. Ambipolar OMIECs however, are scarce, limited by intricate materials design and complex synthesis. Here we demonstrate that judicious selection of p- and n-type materials for blend-based OMIECs offers a simple and tunable approach for the fabrication of ambipolar OECTs and corresponding circuits. These OECTs show high transconductance and excellent stability over multiple alternating polarity cycles, with ON/OFF ratios exceeding 10^3^ and high gains in corresponding inverters. This work presents a simple and versatile new paradigm for the fabrication of ambipolar OMIECs and circuits with little constraints on materials design and synthesis and numerous possibilities for tunability and optimization towards higher performing bioelectronic applications.

## Abstract (web summary)

Ambipolar organic electrochemical transistors simplify bioelectronics circuitry but are challenging due to complicated material design and synthesis. Here, the authors demonstrate that p- and n-type blends offer a simple and tuneable approach for the fabrication of ambipolar devices and circuits.

## Introduction

The detection and amplification of biological signals has been an increasingly important subject of research in the past few decades. For an artificial electronic device to record brain activity^1^, detect ionic biological markers in body fluids^2^, identify organic molecules in tumor cells^3^, etc., it must sense and transmit in real time the changes in an electrochemical potential during a biological process. Coupling the ion-based biological signals to electron-based devices requires materials that support the transport of both ions and electrons, combined with circuit designs that can provide on-site signal amplification. Organic mixed ionic-electronic conductors (OMIECs) are promising in this field because they can couple electronic and ionic currents, are easy to process into circuit architectures and offer mechanical compatibility with soft tissues^4,5^. The most common and versatile device that utilizes OMIECs for the amplification of biological signals is the Organic Electrochemical Transistor (OECT)^6–8^ that relies on the injection/extraction of ions from an electrolyte, regulated by a gate electrode, to modulate the bulk conductivity in an OMIEC channel. The technology for amplification and transfer of the biological signals harnesses the CMOS-like circuit architectures. This technology is based on the layout and wiring of p-type and n-type transistors to perform analog and digital processes. Therefore, both p-type and n-type OMIECs are required to fabricate and wire n-type and p-type OECTs into complex circuits. Most OMIECs, as a subset of organic semiconductors, are however p-type in nature with only a handful of n-types emerging in recent years^9–14^. The design of these n-type OMIECs is usually inspired by semiconducting materials that perform well in n-type Organic Field-Effect Transistors (OFETs), but with sidechains that support ionic transport, yet polymers that bear no side-chains are also a rising class in this group^15^.

OFET technology offers not only p-type and n-type performances for conventional CMOS circuit design, but also ambipolar character, i.e. devices that can act as both p-type and n-type. Ambipolar OFETs, therefore, offer the fabrication of programmable logic circuits with polymorphic functionality^16–18^, and were also shown to significantly reduce the complexity, processing steps and manufacturing costs of complementary-like inverters and logic circuits fabrication, while maintaining reasonably high gains^19–21^. It was very recently shown that ambipolarity in OECTs can also reduce the device footprint and increase device density in circuits, for example through cofacial architecture^22^. In the case of bioelectronics, ambipolarity has another major advantage, the detection of both cations and anions in one device, which further expands the prospects of ambipolar OMIECs as biosensors^23–28^. It was recently found that modification of the glycolated side chains can drive the initially n-type material to perform in a p-type OECTs structure^11^. The p-type character comes on expense of the n-type performance so that the OECT was ambipolar, i.e., the channel can shuttle both holes and electrons between source and drain, but performed quite poorly. A very recent study demonstrated that controlling the dihedral angle and charge delocalization by co-monomer backbone replacement can also transform the OMIEC polarity from p- to n-type^29^. Therefore, the common approach to expand the ambipolar OMIECs material library is the design and synthesis of new materials that involves deep theoretical studies considering the complex interplay between energy level position, chemical structure and resulting film morphology that can support both ionic and electronic transport. This is a challenging route that so far did not lead to the demonstration of well performed and stable ambipolar OECTs, as reviewed in Table 1, and evident from the scarcity of ambipolar OMIECs in previous studies^10,22,30,31^.

**Table 1:** Summary of reported small molecule and ambipolar OECT performances

*Material*	*Structure*	*Polarity*	*V*_*th*_[*V*]	*g*_*m,max*_ [*S cm*^−1^]	*V*_*G*_ *@ g*_*m,max*_ [*V*]	μC^*^ [*F cm*^−1^ *V*^−1^ *s*^−1^)]	Year	Ref.
p(g2T-TT)	Polymer	p	${{0}^e}$	135	−0.5	261	2016	^34^
p(gNDI-gT2)	Polymer	n p	0.35 N/A	0.109 0.067	0.5 −0.8	$0.18$ N/A	2016	^10^
C60-TEG	Small molecule	n	0.556	1.46	0.8	7	2019	^12^
P4E4:PEO^a^	Small molecule: polymer blend	p	−0.15	0.02	−0.47	0.81	2020	^49^
2DPP-OD-TEG^b^	Polymer	n p	0.89 −0.82	0.73 1.65	1.12 −0.97	6.8 31.8	2021	^31^
p(C4-T2-C0-EG)	Polymer	n p	0.3 −0.6	0.031 0.027	0.6 −0.8	0.16 0.13	2021	^22^
p(C4-T2-C0-EG)^c^	Polymer	n p	0.32 N/A	0.31 ~0.3	0.6 −0.85	0.22 ~0.2	2021	^11^
PNDIODTEG-2Tz^d^	Polymer	n p	0.75 N/A	0.117 N/A	0.8 N/A	2.34 N/A	2022	^30^
PNDI2TEG-2Tz^d^	Polymer	n p	0.54 N/A	0.493 N/A	0.8 N/A	1.16 N/A	2022	^30^
4Cl-PDI-4EG	Small molecule	n	0.05	0.0452	0.45	0.13	2022	^60^
4Cl-PDI-3EG	Small molecule	n	0.26	0.0484	0.5	0.17	2022	^60^
PDI-3EG	Small molecule	n	0.34	0.0164	0.5	0.08	2022	^60^
PrC_60_MA	Small Molecule	n	0.620	6.1	0.9	21.7	2022	This work
p(g2T-TT)	Polymer	p	0.001	97.6	−0.3	324.3	2022	This work
PrC_60_MA: p(g2T-TT) 95:5 (w:w)	Small molecule: polymer blend	n p	0.649 −0.09	3.0 4.8	0.9 −0.3	11.8 22.8	2022	This work

^a^Pristine P4E4 had too low a performance to extract $\mu {C}^{*}$ with ${g}_{m} \sim 0.005\left[{Sc}{m}^{-1}\right]$.

^b^Electrolyte used was 0.1 M NaClO_4_.

^c^Study does not report p-type performance, it was extracted visually.

^d^Study does not report p-type performance.

^e^V_ON_ rather than V_TH_.

Inspired by other organic electronic devices, mainly Organic Photovoltaics (OPVs), but also OFETs, we suggest a new design strategy for ambipolar OECTs: blending p- and n-type materials in a single layer, each responsible for the transport of a different charge carrier^32,33^ with a bulk-heterojunction structure. This offers great versatility in material selection as well as excellent control over morphology through processing, without the need to synthesize new materials. In this report we demonstrate, for the first time, blend-based ambipolar OECT devices and corresponding inverters. Building on the vast experience and knowledge gained in OPV research, specifically the insightful understanding of processing-morphology-performance relationships in fullerene:polymer blends, we implemented an analogous blend of judiciously-selected polymer and fullerene-derivative mixed conductors in OECTs. We controlled blend composition, processing conditions and thermal treatments to direct the crystallinity and morphology of the materials in the active layer to support ambipolar transport across the device. These OECTs, as well as the inverters made of two identical ambipolar OECTs, show high current and voltage amplification factors and excellent stability over multiple alternating polarity cycles.

## Results

### Organic electrochemical transistors

**Fig. 1:**
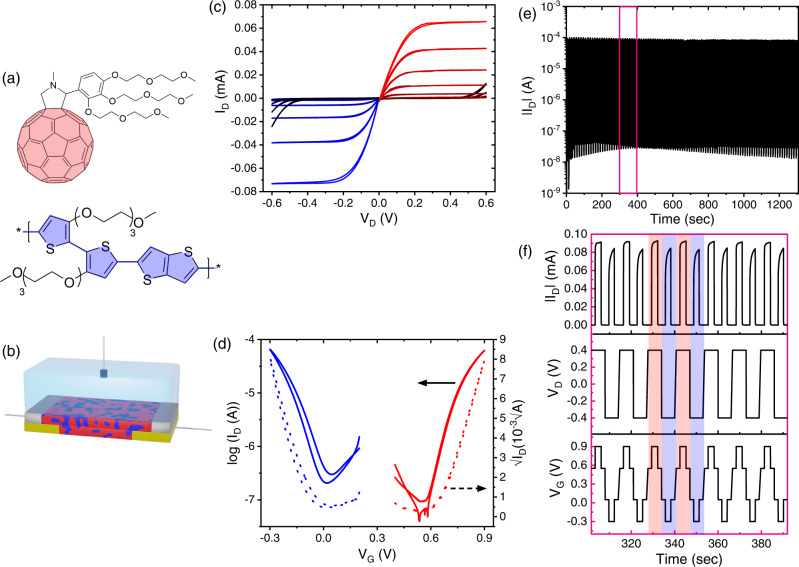
Materials, device characteristics, and long-term stability operation. **a** Chemical structures of the materials used in this study: n-type fullerene derivative PrC_60_MA (top, red) and the p-type polymer p(g2T-TT) (bottom, blue). **b** Schematic of the OECT device with 3-terminals (Au source and drain, Ag/AgCl pellet gate), using a PrC_60_MA:p(g2T-TT) blend as the active channel in a 0.1 M KCl aqueous electrolyte. **c** Output characteristics (I_D_-V_D_) of the ambipolar device (length (L) = 30 $\mu m$, width (W) = 1000 $\mu m$, thickness (**d**) = 60 nm) for V_G_ varying from +0.2 V to −0.3 V at the p-type (black to blue curves in bottom left quadrant) and from +0.4 V to +0.9 V at the n-type (black to red curves in top right quadrant) with a step of 0.05 V. **d** Transfer curves for V_D_ = −0.4 V (p-type, blue) and V_D_ = +0.4 V (n-type, red) in log scale (solid lines) and $\sqrt{{I}_{D}}$ scale (dashed lines). Presented characteristics are averaged over four devices. **e** Typical stability over 100 cycles operation in alternating polarities, each pulsed to ON state for ~3 s with a ~3 s interval time between pulses. **f** Stability measurement of 7 cycles out of total 100 presented in (**e**) (pink rectangle). In the n-type V_G_ is pulsed from +0.55 V to +0.9 V and back to +0.55 V while V_D_ = +0.4 V, while in the p-type V_G_ is pulsed from +0.05 V to −0.3 V and back to +0.05 V while V_D_ = −0.4 V. All of device data presented here was acquired with a blend ratio of 95:5 (w:w) PrC_60_MA:p(g2T-TT).

Our selected p-type component is the poly(2-(3,3′-bis(2-(2-(2-methoxyethoxy)ethoxy)ethoxy)-[2,2’-bithiophen]−5-yl)thieno[3,2-b]thiophene) (p(g2T-TT)) polymer (Fig. 1a, bottom) which has been thoroughly studied for OECTs and its characteristics are well established^34,35^. The n-type fullerene derivative, C_60_,N,N,N-trimethyl-1-(2,3,4-tris(2-(2-methoxyethoxy)ethoxy)phenyl)methanaminium monoadduct (PrC_60_MA, Fig. 1a, top), was “borrowed” from the field of perovskite solar cells and high-k dielectrics^36–38^, and although it was not used in OECTs, a very similar fullerene derivative, C60-TEG, already demonstrated n-type OECT performance^12^. The performances of p(g2T-TT) and C60-TEG are listed in Table 1 for comparison with the current study. In contrast to OPVs where the energy band alignment should encourage charge transfer at the fullerene:polymer interface, here we aimed for a substantial misalignment between the HOMO and LUMO levels to avoid charge transfer at the n:p interface^39^. We speculate that under such conditions the figure of merit for the n-type and p-type performances of the OECT are almost independent, and hence ambipolarity, i.e. balanced performances, can be achieved using a simple rule of mixtures.

Considering the measured ~15-fold higher OECT figure of merit of p(g2T-TT) polymer relative to that of the PrC_60_MA fullerene (Supplementary Table 1, Supplementary Fig. 1) as well as reported values (Table 1)^12,34^, and the high fullerene content required for efficient electron percolation, evident from blend-based ambipolar OFET studies^40–42^, we fabricated OECTs based on PrC_60_MA -rich blends. By comparing the p- and n-type performances for OECTs based on several blend compositions (Supplementary Table 1), we found that similar n and p figures of merit are obtained for a blend ratio of 95:5 w:w. Namely, for this composition the ${{{{{\rm{\mu }}}}}}{{{{{{\rm{C}}}}}}}^{*}$, where *μ* is the carrier mobility and ${C}^{*}$ the volumetric capacitance, are similar for both positive and negative charge carriers. Maintaining the channel dimensions (where W, L and d are channel width, length and thickness, respectively) and the difference between maximum transconductance gate voltage ${{{{{{\rm{V}}}}}}}_{{{{{{\rm{G}}}}}}}$ and threshold voltage ${{{{{{\rm{V}}}}}}}_{{{{{{\rm{th}}}}}}}$ similar for all fabricated OECTs, we can use Eq. 1 to calculate the transconductance (${{{{{{\rm{g}}}}}}}_{{{{{{\rm{m}}}}}}}$) which is directly proportional to ${{{{{\rm{\mu }}}}}}{{{{{{\rm{C}}}}}}}^{*}$.

1 $${{{{{{\rm{g}}}}}}}_{{{{{{\rm{m}}}}}}}=\frac{{{{{{\rm{Wd}}}}}}}{{{{{{\rm{L}}}}}}}{{{{{\rm{\mu }}}}}}{{{{{{\rm{C}}}}}}}^{*}\left|{{{{{{\rm{V}}}}}}}_{{{{{{\rm{th}}}}}}}-{{{{{{\rm{V}}}}}}}_{{{{{{\rm{G}}}}}}}\right|$$

The obtained ambipolar device characteristics display a well-balanced and low-hysteresis behavior, both in output and transfer curves (Fig. 1c, d), with good reproducibility between devices (see Supplementary Fig. 2 and Table 2). It can be noted from Table 2 and Supplementary Table 1 that the threshold voltages of the blend are shifted further away from the OFF voltage, with respect to OECTs based on the pristine materials, indicating that for both polarities doping becomes more difficult upon blending. Interestingly, both the ${{{{{{\rm{g}}}}}}}_{{{{{{\rm{m}}}}}}}$ and ${{{{{\rm{\mu }}}}}}{{{{{{\rm{C}}}}}}}^{*}$ of the p-type performance decrease linearly with p(g2T-TT) content, suggesting that the performance of the polymer is proportional to the number of ion-accessible monomer units in the channel. It has already been demonstrated that semiconducting polymers can be incorporated in blends with extremely low content and still retain mobility in OFETs, i.e. the percolation threshold for charge transport is extremely low^43–45^. Therefore, if the hole mobility in our system is also retained when decreasing the polymer content in the blend, it might suggest that the capacitance decreases with the number of ion-accessible monomer units. On the contrary, the n-type performance is significantly impaired even with the slight decrease in PrC_60_MA concentration in the blend, suggesting that the presence of the long p(g2T-TT) polymer strands obstructs the electron percolation pathways and reduces electron mobility. Comparing the figure-of-merit, ${{{{{\rm{\mu }}}}}}{{{{{{\rm{C}}}}}}}^{*}$, of the blend presented in Table 2 to the best performing materials reveals that although impressive, it still has a long way to reach state-of-the-art p-type (~500 $[{{{{{\rm{F}}}}}}\,{{{{{\rm{c}}}}}}{{{{{{\rm{m}}}}}}}^{-1}{{{{{{\rm{V}}}}}}}^{-1}{{{{{{\rm{s}}}}}}}^{-1}]$)^46,47^ and a small leap for state-of-the-art n-type (~25 $[{{{{{\rm{F}}}}}}\,{{{{{\rm{c}}}}}}{{{{{{\rm{m}}}}}}}^{-1}{{{{{{\rm{V}}}}}}}^{-1}{{{{{{\rm{s}}}}}}}^{-1}]$)^15^.

**Table 2:** Summary of blend-based OECT characteristics

Blend Ratio		Polarity	*g*_*m,max*_ [*S cm*^−1^]^*a*^	μC^*^ [*F cm*^−1^ *V*^−1^ *s*^−1^]^*b*^	*V*_*th*_ [*V*]^*c*^
**95:5**					
	PrC_60_MA	n	3.0 ± 0.6	11.8 ± 1.4	0.649 ± 0.018
	p(g2T-TT)	p	4.8 ± 0.2	22.8 ± 0.9	−0.090 ± 0.003

^a^Calculated from transfer curves according to ${g}_{m}=\frac{\partial {I}_{D}}{\partial {V}_{G}}$ divided by $\frac{{Wd}}{L}$

^b^Calculated with $W=1000\mu m,L=30\mu m,d=60-70{nm}$ according to Eq. 1.

^c^Calculated with linear fit of $\sqrt{{I}_{D}}$ *vs V*_*G*_ at V_D_ = ± 0.4 V

The high fullerene content in our blend has been a subject for concern in terms of device stability, as the reduced fullerene species with an adjacent metal cation may form a soluble product^48^. This was demonstrated in a recent study on C_60_-TEG that found that the a C_60_-TEG film quickly degrades over repeated cycling^12^. However, in our case, after 100 cycles in alternating polarities we noticed no significant current degradation and the current ON/OFF ratios that were extracted from the stability measurements remained above 10^3^ (Fig. 1e, f). A previous study on small molecule p-type OECTs blended with high molecular weight PEO showed a similar trend of improved film stability over cycles upon blending, yet an explanation was not provided^49^. Examining the current for both polarities reveals that the p-type transient response time, ${{{{{{\rm{\tau }}}}}}}_{{{{{{\rm{ON}}}}}}}=466\pm 56{{{{{\rm{ms}}}}}}$, is slower than that of the n-type, ${{{{{{\rm{\tau }}}}}}}_{{{{{{\rm{ON}}}}}}}=20\pm 3{{{{{\rm{ms}}}}}}$, by a factor of ~25 (Supplementary Table 2, Supplementary Fig. 3). These differences are attributed to the low content of the polymer in the film, making the pathways for anion injection and doping scarcer. This correlates well with the slightly higher hysteresis of the device characteristic in p-type operation compared to n-type operation (blue and red curves in Fig. 1d, respectively). Notably, the p-type response time of the ambipolar OECT is slower than that of unipolar OECTs based on the same material, probably due to the very small amount of the p-type polymer in the blend. In contrast, the n-type response time of the ambipolar OECT is the fastest reported for small molecule n-type OECTs although it is blended with the polymer.

### Electrochemical and optical properties

**Fig. 2:**
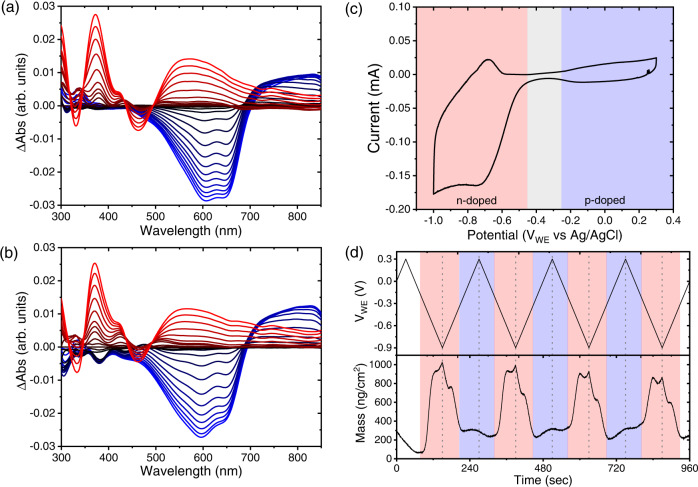
Electrochemical and optical properties. Steady-state spectroelectrochemical absorption change of the ambipolar films (**a**) measured in the 95:5 blend, and **b** calculated from a rule of mixtures of the steady-state spectroelectrochemical absorption change of the pristine materials (Supplementary Fig. 4). For all measurements the voltage is applied vs an Ag/AgCl pellet electrode at a range of + 0.3 V < V_WE_ < −0.9 V with steps of 0.05 V. Absorption changes are displayed with respect to the recorded spectrum at V_WE_ = −0.4 V. Blue curves denote changes that occur as a result of spectral changes in p(g2T-TT), whereas red curves originate from spectral changes in PrC_60_MA. Formation of the positive polymer polaron is visible in the blue curves peak at ~830 nm, rising on the expense of the $\pi -{\pi }^{*}$ interactions peaks at 600–650 nm^34^. In the PrC_60_MA red curves, the broad absorption change is due to the formation of a radical anion that stretches into the NIR region^61,62^. As the HOMO of p(g2T-TT) and LUMO of PrC_60_MA are ~0.7 eV apart (Supplementary Fig. 6), the changes in absorption for each of the components are independent and spectrally resolved. This is also manifested in **c** Cyclic voltammetry of the ambipolar 95:5 blend in an EQCM cell at 0.1 V/sec, showing the p- and n-doped current as well as a “neutral” zone where there is no doping. **d** Mass changes in the 95:5 blend while performing CV in an EQCM cell at 0.01 V/sec, red areas belong to PrC_60_MA response with injection/expulsion of K^+^ cations, and blue to p(g2T-TT) response with injection/expulsion of Cl^-^ anions.

The ambipolar nature of the selected blend in also reflected in steady-state spectroelectrochemistry (SEC) measurements recorded in the same voltage range as the OECTs. Taken at similar thicknesses, spectral changes are calculated via a simple rule-of-mixtures according to the weight percent of each material, and compared to the measured blend (Fig. 2a, b). The changes in the absorption spectrum of the blend as a function of applied voltage corroborate the doping of both p- and n-type components and show that the measured changes comply well with the calculated, namely the spectroelectrochemical changes are additive. Compared to the calculated spectrum, both $\pi -{\pi }^{*}$ and polaronic peaks of p(g2T-TT) exhibit a slight red shift in the measured spectra vs the calculated, and a higher A_0-0_/A_0-1_ vibronic peaks ratio (Supplementary Fig. 5). Together, these observations suggest a higher degree of polymer J-aggregation in the blend than in the pristine film^50,51^, namely, the polymer adopts a more fibrous structure in the blend. A possible explanation is that the p(g2T-TT) strands in the blend are surrounded by the less polar fullerene matrix that induces more J-aggregation. A similar observation was reported for polythiophene co-polymers with polar glycolated sidechains where J-aggregation increases when polarity is reduced^52^.

Cyclic Voltammetry (CV) confirmed the electrochemical activity of both species in the blend, with three distinguishable regions for n-doping, p-doping and film neutrality (Fig. 2c). Electrochemical Quartz Crystal Microbalance (EQCM) measurements demonstrated the expected mass gain on both polarities, within the same voltage range as the OECTs, SEC and CV measurements (Fig. 2d). Notably, the mass gain on the p-type is notably lower than on the n-type (~100 vs ~700 $[{{{{{\rm{ng}}}}}}/{{{{{{\rm{cm}}}}}}}^{2}]$), as a result of the significantly lower content of the polymer in the blend. To corroborate the assignment of the low mass gain on the p-type to chloride injection/extraction, we compared it to the EQCM responses of pristine material and a blend with higher polymer content (Supplementary Figs. 7, 8). Thus, the injection and expulsion of both anions and cations as doping agents in the same film is demonstrated here via EQCM for the first time, to the best of our knowledge. The overall spectroelectrochemical and electrochemical results imply that not only there is no chemical interaction between the blended materials, but also that the steady-state doping of each material is mostly unaffected by the presence of the other, as hypothesized by us at the materials selection stage. It is worth noting that the reduction current of PrC_60_MA consists of a large Oxygen Reduction Reaction (ORR) contribution^53^ as a result of the relatively shallow LUMO level. The simple decoupling between p-type and n-type signals is a result of the significant energy gap between PrC_60_MA LUMO and p(g2T-TT) HOMO and opens possibilities for independent tunability.

### Microstructural characterization

**Fig. 3:**
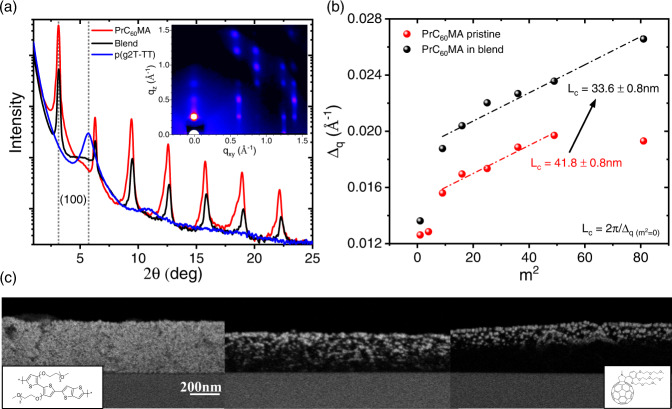
Structural characterization. **a** Out-of-plane XRD of pristine materials and the 95:5 w:w PrC_60_MA:p(g2T-TT) blend and the Grazing Incidence Wide-Angle X-ray Scattering (GIWAXS) measurement of the blend (inset). Dotted lines at 2*θ* = 3.1° and 5.7° are associated with the (100) peaks of PrC_60_MA and p(g2T-TT), respectively. All other peaks are higher (h00) reflections of PrC_60_MA. **b** Scherrer analysis of the coherence length (L_c_) of PrC_60_MA crystals in pristine and 95:5 blend films using a pseudo-voigt function fitting. In this analysis the FWHM of each (h00) reflection, ${\triangle }_{q}$, is plotted vs the square reflection order, m^2^, and the extrapolation to the 0^th^ order provides the L_c_^55,63^ (Supplementary Fig. 10). **c** Back-Scattered Electrons (BSE) detector cross-section High Resolution Scanning Electron Microscopy (HRSEM) images of the p(g2T-TT) (left), 95:5 blend (middle), and PrC_60_MA (right) films following a Vapor Phase Infiltration (VPI) ‘staining’ process. The bright contrast is due to nucleation and growth of ZnO particles. The distribution of ZnO particles throughout the blend film, in contrast to the pristine PrC_60_MA film, corroborates the bulk-heterojunction morphology. The scale bar is 200 nm.

To gain understanding on the correlation between blend morphology and device performance we performed structural characterization. Grazing Incidence Wide Angle X-ray Scattering (GIWAXS) measurements indicate that the blend film is highly crystalline with a strong preferential orientation relative to the substrate, similarly to neat PrC_60_MA films (Supplementary Fig. 9) and other fullerene derivatives^12,54^, though it is significantly less crystalline than the neat PrC_60_MA film (Fig. 3a). While the lamellar stacking d-space is practically unchanged (Table 3), it is found that the out-of-plane coherence length of PrC_60_MA is reduced by a staggering 20% (Fig. 3b, Supplementary Fig. 10). This decrease in coherence length and crystallinity lowers the mobility and overall performance of the semiconductor in the blend^55^ leading to the obtained two-fold reduction of the PrC_60_MA figure of merit upon blending with 5 wt% polymer only.

**Table 3:** Summary of out-of-plane XRD characteristics

Material		Lamellar stacking q [Å^−1^] ^a^	Lamellar stacking d [Å] ^a^
**Pristine**			
	p(g2T-TT)	0.4071	15.44
	PrC_60_MA	0.2234 ± 0.0002	28.13 ± 0.03
**95:5 Blend**			
	p(g2T-TT)	-^b^	- ^b^
	PrC_60_MA	0.2248 ± 0.0004	27.96 ± 0.06

^a^Calculated from an average of 7–8 peaks in out-of-plane XRD, for p(g2T-TT) only one peak is observed.

^b^Could not extract peak reliably from spectrum due to convolution with the (200) peak of PrC_60_MA.

As a complementary tool to GIWAXS, pristine and blend films were exposed to a Vapor-Phase Infiltrated (VPI) process of diethyl zinc (DEZ) and water which to reveal the nanoscale morphology via High Resolution Scanning Electron Microscopy (HRSEM). Visualization of different phases in organic films is challenging, however in VPI the ZnO precursors selectively infiltrate different phases, resulting in a Z-contrast ‘staining’ detectable by cross section HRSEM. Figure 3c shows that ZnO precursors easily infiltrate p(g2T-TT) films, evident from the strong bright contrast, but generally accumulated on the surface of PrC_60_MA films with limited infiltration. The cross section HRSEM image of the blend film shows presence of ZnO particles evenly distributed throughout the film. This measurement confirms that the nanoscale morphology of the ambipolar layer is indeed a uniform bulk-heterojunction-like, and not, for example, a phase-separated bilayer^56^. Moreover, the visible interpenetrating network agrees with the reduction of coherence length of PrC_60_MA alongside retainment of connectivity between p(g2T-TT) chains.

### Inverter logic circuit

**Fig. 4:**
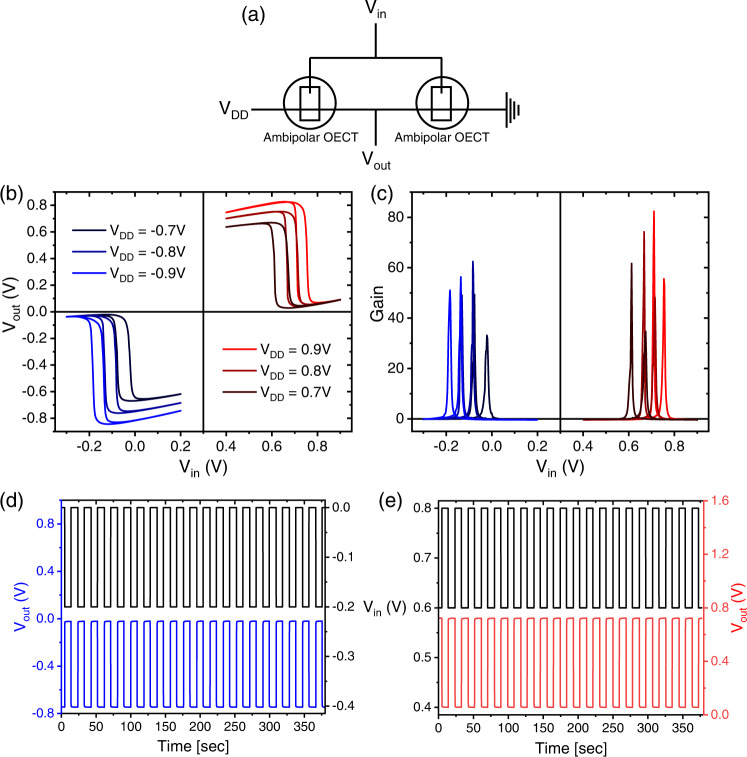
Ambipolar-based Inverter characteristics. **a** Wiring diagram of an inverter consisting of two identical ambipolar OECTs connected in series, one grounded and the other connected to V_DD_, with a common gate electrode applying V_in_ and an output voltage V_out_. The range for V_in_ was selected to be the same as V_G_ of the blend OECT presented in Fig. 1. **b** Voltage Transfer Characteristics (VTC), showing activity in both quadrants centering around V_in_ = +0.3 V at a voltage scan rate of 0.1 V/sec; small hysteresis of ~0.05 V is observed in all scans. **c** Gains calculated from the slope of the VTC, with forward and back peaks of 62, 35 (V_DD_ = 0.7 V); 74, 48 (V_DD_ = 0.8 V); 82, 56 (V_DD_ = 0.9 V); 33, 49 (V_DD_ = −0.7 V); 62, 49 (V_DD_ = −0.8 V); 56, 51 (V_DD_ = −0.9 V). **d**, **e** Shows a 20-cycle response of the inverter to input voltage square pulses for V_DD_ = −0.8 V and + 0.8 V, respectively. All of device data presented here was acquired with a blend ratio of 95:5 (w:w) PrC_60_MA:p(g2T-TT).

Finally, to demonstrate the feasibility of the symmetrical performance for circuitry we fabricated inverters (NOT logic gates) using two identical ambipolar OECTs (Fig. 4a). Applying both positive and negative ${{{{{{\rm{V}}}}}}}_{{{{{{\rm{DD}}}}}}}$, we were able to measure the voltage transfer characteristics (VTC) of the inverter in both quadrants (Fig. 4b), showing small hysteresis ($\Delta {{{{{{\rm{V}}}}}}}_{{{{{{\rm{in}}}}}}}=0.05{{{{{\rm{V}}}}}}$) that suggests a small overpotential is required to turn off the OECTs. This might be due to the different energetic barriers for injection and expulsion of the two ions, or possibly due to different ion kinetics. Since the operating voltages of this blend system are symmetrical around ${{{{{{\rm{V}}}}}}}_{{{{{{\rm{G}}}}}}}=0.3{{{{{\rm{V}}}}}}$ (Fig. 1d) rather than 0 V, the indication for a well-balanced ambipolar inverter is that the voltage step occurs at $\left(\frac{{{{{{{\rm{V}}}}}}}_{{{{{{\rm{DD}}}}}}}}{2}+0.3{{{{{\rm{V}}}}}}\right)$. Very high gains are calculated from the VTC (Fig. 4c), slightly lower than those reported for the state-of-the-art OECT inverters^15,57^ but still outperforming most^13,22,31,58,59^. Stability of the inverter is also recorded over 20 cycles and found to be excellent (Fig. 4d, e). It is important to note that while processing becomes simpler in ambipolar inverters, it is not free of problems compared to traditional CMOS inverters. The fact that ambipolar transistors cannot be totally switched off causes a Z-shape in the VTC which, in turn, is translated to higher static power consumption and lower static noise margins. Still, the improved performance and stability of the system reported here, and its unique tunability possibilities open new directions for exploiting ambipolar blend-based OECT circuitry.

## Discussion

We have demonstrated the bulk-heterojunction methodology as a viable and promising tool in the ambipolar OECTs toolbox that opens exciting new venues with a broad matrix of tunable parameters and relationships to be unraveled. We show, for the first time, an ambipolar OECT based on a blend of two materials with opposite polarities, a fullerene derivative and a polythiophene, with improved performances compared to current ambipolar OMIECs. Our study shows that not only both components are optically, electrochemically and electrically active, but also do not interfere with one another. We demonstrate the utilization of this blend in a single OECT as well as an inverter made of two identical OECTs, easily fabricated using a single solution-processing step. Importantly, film integrity and electrical stability are very good due to the bulk-heterojunction morphology with the interpenetrating network. A main advantage of the blend approach over the synthesis of new ambipolar materials is that each component can be independently optimized, overcoming the built-in p-n trade-off that is inherent to a single ambipolar material. Moreover, the continuously growing library of OECT materials offers multiple combinations and possibilities to tune and improve the OECT performances. We suggest that when selecting the blend components, one should consider a few aspects. First, from the morphology point of view, material phase separation and the interplay between amorphous and crystalline domains should be controlled and optimized to support the transport of both ionic and electronic charge carriers. Second, both components should attain similar performances so that blend composition could be relatively balanced. Third, the threshold voltages (determined by the energy levels) should be symmetrical around 0 V so that the device can serve as a building block in advanced CMOS-like circuitry. Finally, we expect the ambipolar OECT to pave the way toward the design and fabrication of a new generation of simply-processed, compact and dynamically reconfigurable advanced bioelectronic devices as well as biologic sensors with dual detection of cations and anions for bodily fluids and soft tissues.

## Methods

### Materials

Poly(2-(3,3′-bis(2-(2-(2- methoxyethoxy)ethoxy)ethoxy)-[2,2’-bithiophen]−5-yl)thieno [3,2-b]thiophene) (p(g2T-TT), Mn = 8.1 kDa, Mw = 18.3 kDa from GPC in DMF at 40 ^o^C) was synthesized by the McCulloch group according to previous reports and used as received^34^. C_60_,N,N,N -trimethyl-1-(2,3,4-tris(2-(2-methoxyethoxy)ethoxy)phenyl)methanaminium monoadduct (PrC_60_MA, Solenne BV), KCl (Sigma-Aldrich Israel Ltd.), Diethylzinc (packaged for use in deposition systems, Sigma-Aldrich Israel Ltd.) were used as received.

### Film Preparation

All substrates were cleaned prior to film deposition in an ultrasonic bath in acetone, methanol and isopropanol (15 min each) and blow-dried in N_2_ (99.995%). p(g2T-TT) and PrC_60_MA were dissolved in chloroform (anhydrous, contains amylene as stabilizer, ≥99%, Sigma-Aldrich Israel Ltd.) at concentrations of 5 mg/ml and either 10 mg/ml or 30 mg/ml at room temperature, respectively. For blend preparation, pristine material solutions were mixed in the appropriate amounts to achieve 90:10, 95:5 and 99:1 (w:w) blend ratios. All solutions were spin-coated at room temperature onto substrates at 1000 rpm for 60 s followed by 3000 rpm for 10 s, to obtain ~60–70 nm film thicknesses, when using 5 mg/ml p(g2T-TT), 10 mg/ml PrC_60_MA and 9.5 mg/ml of the blend. For VPI measurements we prepared thicker films, ~175 nm, using 5 mg/ml p(g2T-TT) with 500 rpm for 60 s, 30 mg/ml PrC_60_MA with 1000 rpm for 60 s, and 24 mg/ml blend with 1000 rpm for 60 s. All films were subsequently annealed on a hot plate at 120 ^o^C for 20 min. Film preparation was executed entirely under inert atmosphere inside a glovebox. Film thicknesses were measured using a stylus profilometer (Bruker DektakXT) with a $12.5\mu m$ radius of the needle tip.

### Spectroelectrochemistry (SEC) and Cyclic Voltammetry (CV)

Fluorine-doped tin oxide (FTO) coated glass (surface resistivity ~7 Ω/sq, Sigma-Aldrich Israel Ltd.) was used as a conductive transparent WE and coated with materials as described above. The FTO-coated substrates were dipped in a 0.1 M KCl solution in a glass cuvette (3/G/10, Starna Scientific Ltd.) with a Pt wire as CE and a Ag/AgCl pellet (E206 Warner Instruments, LLC) RE. The setup was put inside a UV-Vis spectrophotometer (Cary 100 Scan, Agilent Technologies, Inc.) and connected to an external potentiostat (Palmsens4, PalmSens BV) as a controller. To calculate the expected spectrum of the blend a rule of mixtures was applied according to the equation:

$${Ab}{s}_{{calc}}=0.95\cdot {Ab}{s}_{{\Pr }{C}_{60}{MA}}+0.05\cdot {Ab}{s}_{p\left(g2T-{TT}\right)}$$

### Electrochemical quartz-crystal microbalance (EQCM)

Gold-coated quartz crystal sensors (5 MHz for Q-sense E1/E4, RenLux Crystal Ltd.) was used as WE and coated with materials as described above. The sensor was placed in a dedicated cell (QWEM 401, Q-sense Explorer, Biolin Scientific) with a Pt wire CE and Ag/AgCl (3 M KCl) RE. The sensor was put in the module and aqueous 0.1 M KCl solution was continuously pumped into the chamber at a flow rate of 100 μl/min. CV in the range + 0.3 V to −0.9 V was performed for several cycles at a scan rate of 10 mV/sec and the frequencies recorded over 7 overtones. As the films are thin they were treated as rigid, therefore we could use the Sauerbrey equation to convert Δf/n to the change in areal mass Δm:

$$\triangle m=-C\cdot \frac{\triangle f}{n}=-5.9\cdot \triangle f\left[\frac{{ng}}{c{m}^{2}}\right]$$

This equation uses $C=17.7 \big[\frac{{ng}}{c{m}^{2}\cdot {Hz}}\big]$ as a constant for a 5 MHz crystal, a constant that is dependent on the wave velocity in quartz plate, the density of quartz and the fundamental resonance frequency. Only the 3^rd^ overtone was used in calculations; therefore, it is put into the equation as well as $n=3$.

### Vapor Phase Infiltration (VPI)

Vapor Phase Infiltration (VPI) process of Zinc Oxide into the organic films was performed in an Atomic Layer Depositions system (Savannah® S200, Veeco Instruments Inc.). Silicon substrates were cleaned followed by film deposition via spin-coating as described above. The samples were then put in the ALD system and exposed to Diethylzinc vapors and DI water in a pulse-exposure-purge sequence. The cycle was repeated 80 times, with N_2_ (99.99%) as the carrier and purging gas. The reaction chamber was heated to 60 ^o^C and the base flow rate of the reactor was 20 sccm.

### High Resolution Scanning Electron Microscopy (HRSEM)

High-Resolution Scanning Electron Microscopy (HRSEM) cross-section images of films on Silicon substrates were recorded using a Zeiss Ultra-Plus FEG-SEM at 1.5 keV, with a Back-scattered Electrons (BSE) detector for Z-contrast observation between ZnO grains and unstained organic regions in the film.

### X-ray Diffraction (XRD)

Out-of-plane $\theta /2\theta$ XRD measurements of films on silicon substrates were recorded using a Rigaku SmartLab 9 kW x-ray diffractometer with D/tex Ultra250 0D detector and Cu Kα radiation (λ = 1.5418Å). The conversion of $2\theta$ to d-space was performed using regular bragg’s equation:

$$n\lambda=2d\cdot {{\sin }} \, \theta$$

To calculate the coherence length, we applied a pseudo-voigt function fitting on each peak of PrC_60_MA (in both the pristine and blend films). The fitting function is of the form of a linear combination of a Gaussian and a Lorentzian with a similar FWHM:

$$I={I}_{0}+A\left[{m}_{u}\frac{2}{\pi }\frac{{{{{{\boldsymbol{w}}}}}}}{4{\left(q-{q}_{c}\right)}^{2}+{{{{{{\boldsymbol{w}}}}}}}^{2}}+\left(1-{m}_{u}\right)\frac{\sqrt{4{{{{{\rm{ln}}}}}}2}}{w\sqrt{\pi }}{e}^{-\frac{4{{{{{\rm{ln}}}}}}2}{{{{{{{\boldsymbol{w}}}}}}}^{2}}{\left(q-{q}_{c}\right)}^{2}}\right]$$

where $I$ is the overall intensity, ${I}_{0}$ offset intensity, A the area, ${m}_{u}$ profile shape factor, $q$ the scattering vector, ${q}_{c}$ scattering vector peak center, and w the FWHM of the peak. Since finite grain size and cumulative disorder is common in organic films, diffraction peaks that belong to the same set of crystal planes, namely (h00), displayed a successive broadening at higher orders. The extracted FHWM was plotted against the square peak order m^2^ and the extrapolated intersection at m^2^ = 0 was taken as $\frac{2\pi }{{L}_{c}}$, where ${L}_{c}$ is the coherence length, following a method of analysis described elsewhere^55^.

For 2D-GIWAXS measurements we used Rigaku SmartLab 9 kW x-ray diffractometer with Cu Kα radiation (λ = 1.54186Å), equipped with a HyPix-3000 2D detector and an aperture slit installed. Incident angle was $\omega=0.2^\circ$.

### Organic Electrochemical Transistors (OECTs) device fabrication and measurement

Ultra-flat quartz-coated glass 15 mm x 20 mm substrates were cleaned as described above and blow-dried in N_2_ (99.995%). Then, substrates were placed in an OFET shadow mask (E291, Ossila Ltd) with $W=1000\mu m,L=30\mu m$. The assembly was put inside a metal thermal evaporator (Edwards Auto 500), where 5 nm chromium and 50 nm gold were evaporated at a pressure of ~6E-6 Torr. Substrates were additionally cleaned for 15 min in isopropanol in an ultrasonic bath, blow dried with N_2_ gun and coated with the organic film. The film was dry-wiped from most of the metal contacts except for the channel area to minimize device crosstalk. Finally, Kapton® tape was used to manually mask the areas where the metal contacts were exposed to avoid contact between electrolyte and metal.

For OECT device characterization, a dual-channel source measure unit (SMU) (B2902A, Keysight Technologies, Inc.) was used with a dedicated software (EasyExpert group+). The SMU was connected to source & drain gold contacts as well as Ag/AgCl pellet (E206 Warner Instruments, LLC) that was used as the gate electrode. The electrolyte was 0.1 M KCl and dropped onto the devices prior to measurement. Output and Transfer curves were acquired with a double-sweep measurement. Calculations were based on an average of 4 devices per composition, each device averaged over 3 operation cycles, using a custom-made MATLAB script. The threshold voltage ${V}_{{th}}$ was calculated by using a linear fit of $\sqrt{{I}_{D}}$ vs V_G_ from the transfer curves and extraction of the intercept point between the fit and $\sqrt{{I}_{D}}=0$. Transconductance and $\mu {C}^{*}$ were calculated from transfer curves at maximum V_G_ for each polarity ($-0.3V$ for p-type, $+0.9V$ for n-type) according to the equation:

$${g}_{m}=\frac{\partial {I}_{D}}{\partial {V}_{G}}=\frac{{Wd}}{L}\mu {C}^{*}\left|{V}_{{th}}-{V}_{G}\right|$$

For stability measurements the same setup as OECT characterization was used, yet square wave pulses were applied instead of regular sweep. The pulses were applied in another dedicated software (Quick I/V Measurement).

For inverter measurements 2 transistors were connected in series at the probing pads with silver paste, channel 1 was connected to V_in_ and ground, channel 2 connected to V_out_, and another SMU (2612B, Keithley Instruments, Inc.) connected to V_DD_. The voltage transfer characteristics were recorded at a rate of 0.1 V/s, 5 cycles for each curve, using Quick I/V Measurement software. The gain was calculated as $\frac{\delta {V}_{{out}}}{\delta {V}_{{in}}}$ from voltage transfer characteristics.

Response time of the ambipolar 95:5 blend was extracted from the stability measurement using an exponential fit:

$${I}_{D}={I}_{D.0}+A\cdot {{\exp }}\left(-\frac{t}{\tau }\right)$$

## Supplementary information

Supplementary Information

## Data Availability

The authors declare that the data supporting the findings of this study are available within the paper and its supplementary information files.
