# Supplementary material for: Ambipolar blend-based organic electrochemical transistors and inverters
Source: Nat Commun. 2022 Sep 22;13:5548. doi: 10.1038/s41467-022-33264-2 (PMC9500051; doi:10.1038/s41467-022-33264-2)
Supplement: Supplementary file 1 — Supplementary Information [file 41467_2022_33264_MOESM1_ESM.pdf]

Supplementary Information

Ambipolar Blend-based Organic Electrochemical Transistors and Inverters

Eyal Stein[1], Oded Nahor[1], Mikhail Stolov[2], Viatcheslav Freger[2], Iuliana Maria Petruta[3], Iain McCulloch[3,4] and Gitti L. Frey[1,].*

Supplementary Table 1 | Summary of OECT characteristics for pristine materials and other blend compositions

Material	Polarity	$g_{m,max}[S\ cm^{-1}]$ [a]	$\mu C^*[F\ cm^{-1}V^{-1}s^{-1}]$ [b]	$V_{th}\ [V]$ [c]
**Pristine**				
$PrC_{60}MA$	n	6.1 ± 0.5	21.7 ± 1.6	0.620 ± 0.005
p(g2T-TT)	p	97.6 ± 5.2	324.3 ± 17.7	0.001 ± 0.003
**90:10**				
$PrC_{60}MA$	n	2.5 ± 0.5	8.8 ± 1.4	0.617 ± 0.009
p(g2T-TT)	p	11.4 ± 1.4	45.8 ± 7.2	-0.049 ± 0.014
**99:1**				
$PrC_{60}MA$	n	2.6 ± 0.4	10.3 ± 1.4	0.645 ± 0.014
p(g2T-TT)	p	0.012 ± 0.014	0.023 ± 0.018	- [d]

a) Calculated from transfer curves according to $g_m = \frac{\partial I_D}{\partial V_G}$ divided by $\frac{Wd}{L}$

b) Calculated with $W = 1000\mu m, L = 30\mu m, d = 60nm$ according to $g_{m,max} = \frac{Wd}{L}\mu C^*|V_{th} - V_G|$

c) Calculated with linear fit of $\sqrt{I_D}\ vs\ V_G$ at V_D=±0.4V

d) Cannot be extracted reliably from the results

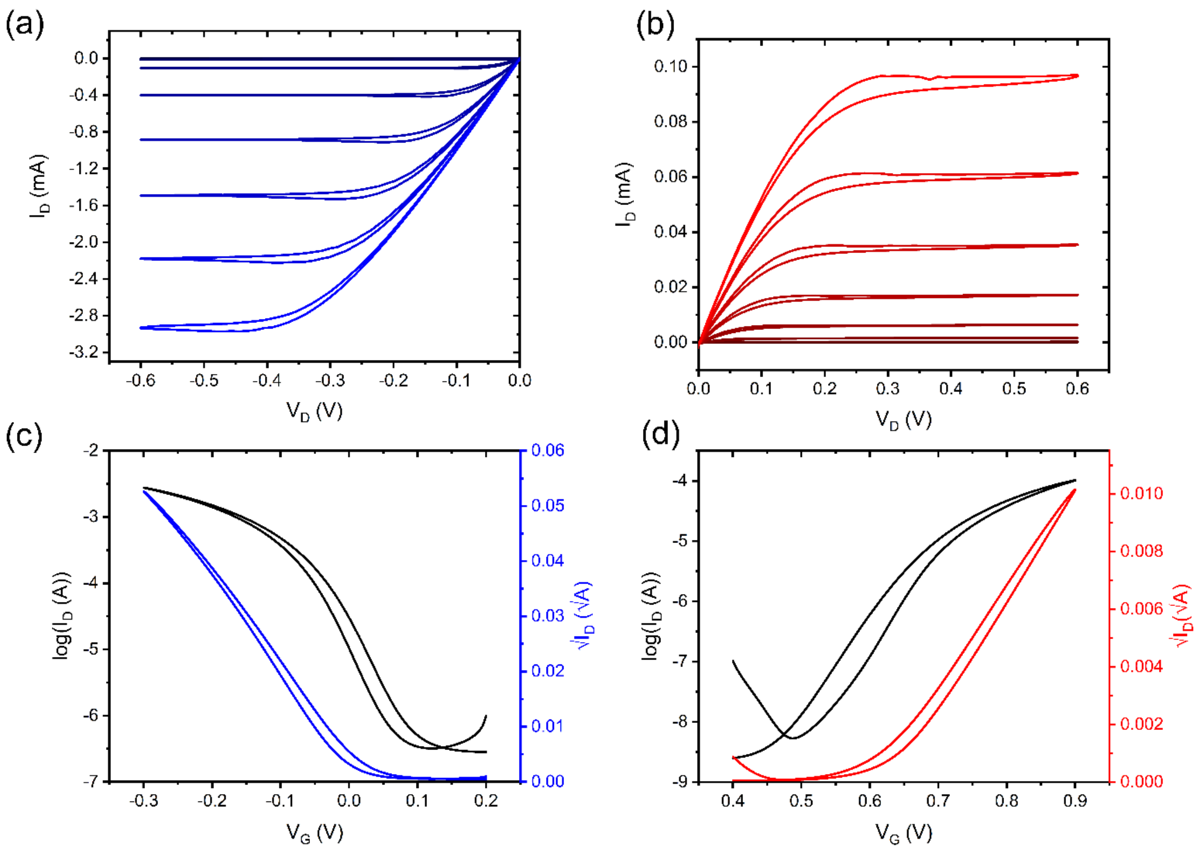

Supplementary Figure 1 | OECT characteristics of pristine materials. (a), (b) Output and (c), (d) Transfer characteristics of pristine p(g2T-TT) and $PrC_{60}MA$, respectively.

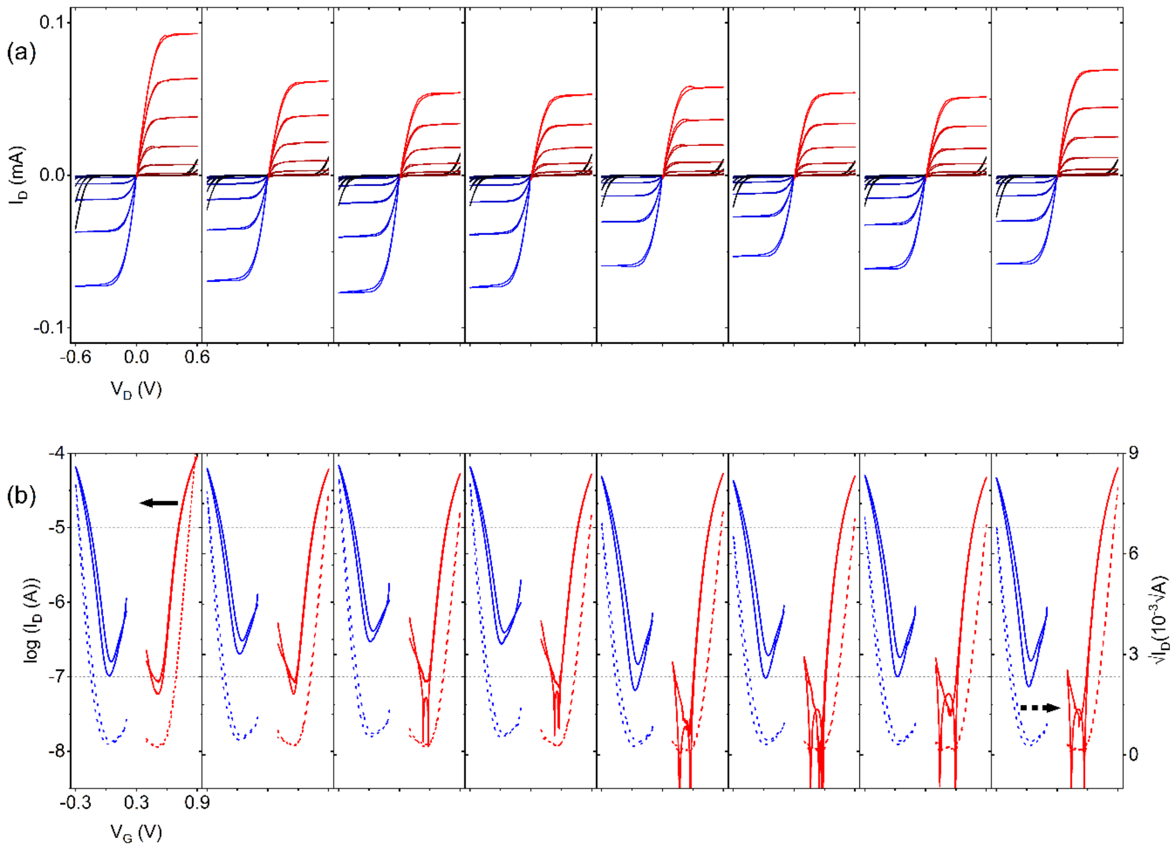

Supplementary Figure 2 | Reproducibility of OECT characteristics of the 95:5 blend. Displayed are (a) output and (b) transfer characteristics of 8 different devices.

Supplementary Table 2 | The transient response time of an ambipolar 95:5 blend OECT extracted from the data resented in Supplementary Figure 3

	Sampling 1	*Sampling 2*	*Sampling 3*	*Average*
n − type				
τ_{ON} [ms]	22.7 ± 0.4	19.9 ± 0.5	17.8 ± 0.4	20 ± 3
τ_{OFF} [ms]	5.56 ± 0.06	5.58 ± 0.07	5.37 ± 0.08	5.6 ± 0.1
p − type				
τ_{ON} [ms]	502 ± 19	401 ± 17	494 ± 23	466 ± 56
τ_{OFF} [ms]	5.6 ± 0.1	6.3 ± 0.1	5.5 ± 0.1	5.8 ± 0.4

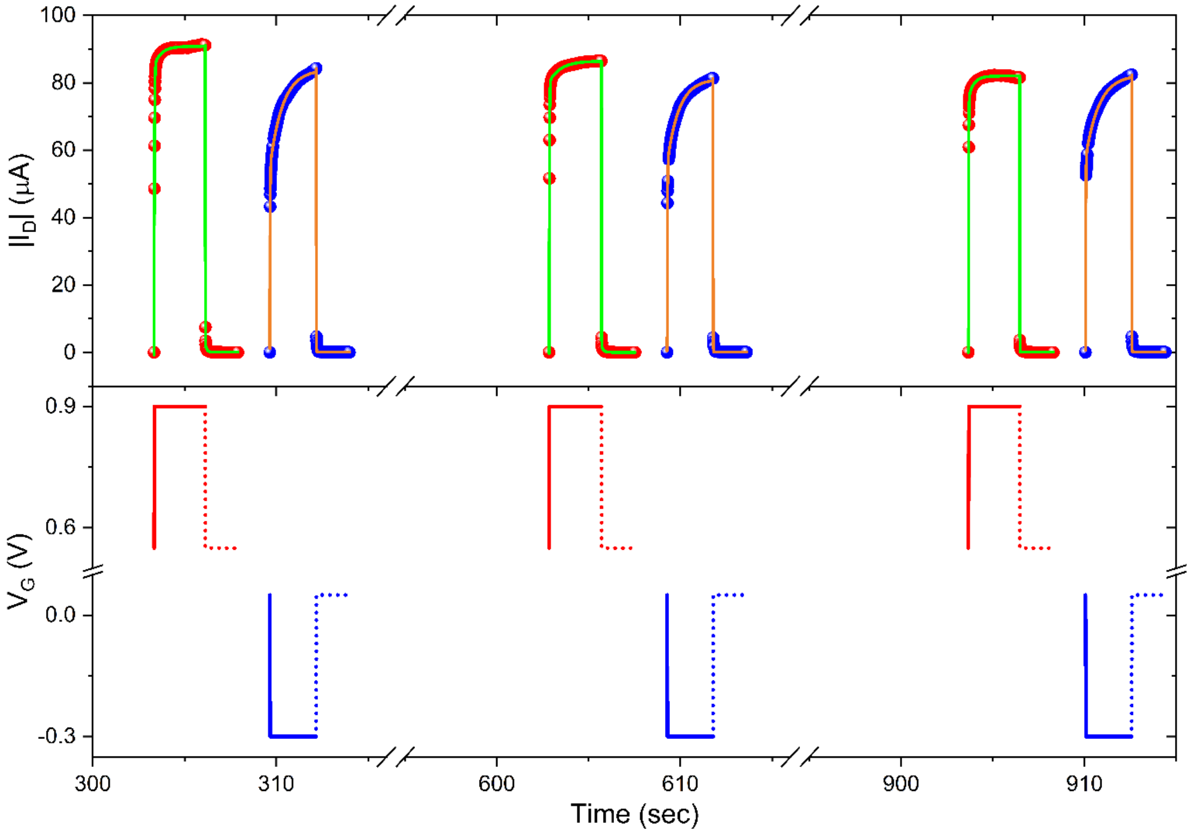

Supplementary Figure 3 | Transient response time analysis of the ambipolar 95:5 blend OECT, with exponential fit curves.

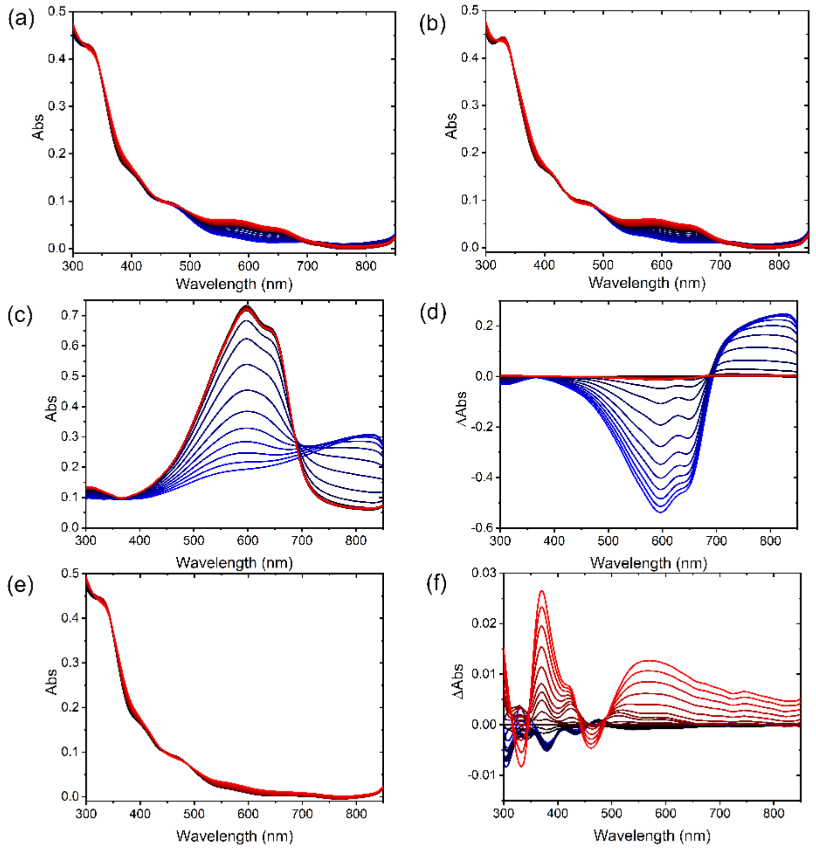

Supplementary Figure 4 | Spectroelectrochemical properties of materials and blend. (a) Rule-of-mixtures calculated and (b) measured absorption spectra for 95:5 w:w $PrC_{60}MA$:p(g2T-TT) blend. (c) Absorption and (d) absorption changes in pristine p(g2T-TT). (e) Absorption and (f) absorption changes in pristine $PrC_{60}MA$. The potentials applied are $-0.9V<V_{WE}<+0.3V$ vs Ag/AgCl pellet. Absorption changes are with respect to the recorded $V_{WE}=-0.4V$ curve.

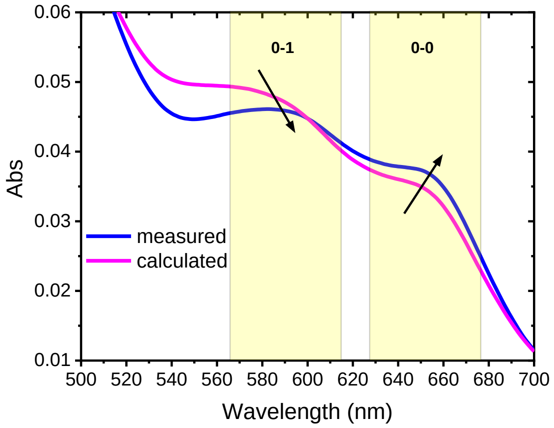

Supplementary Figure 5 | Vibronic absorption peaks. Absorption in the $\pi - \pi^*$ peaks of p(g2T-TT) at -0.4V applied between the film-coated FTO WE and an Ag/AgCl pellet RE with a Pt wire as CE. A notable red-shift is observed in the 0-0 and 0-1 peaks, as well as an increase in the $A_{0\text{-}0}/A_{0\text{-}1}$ ratio.

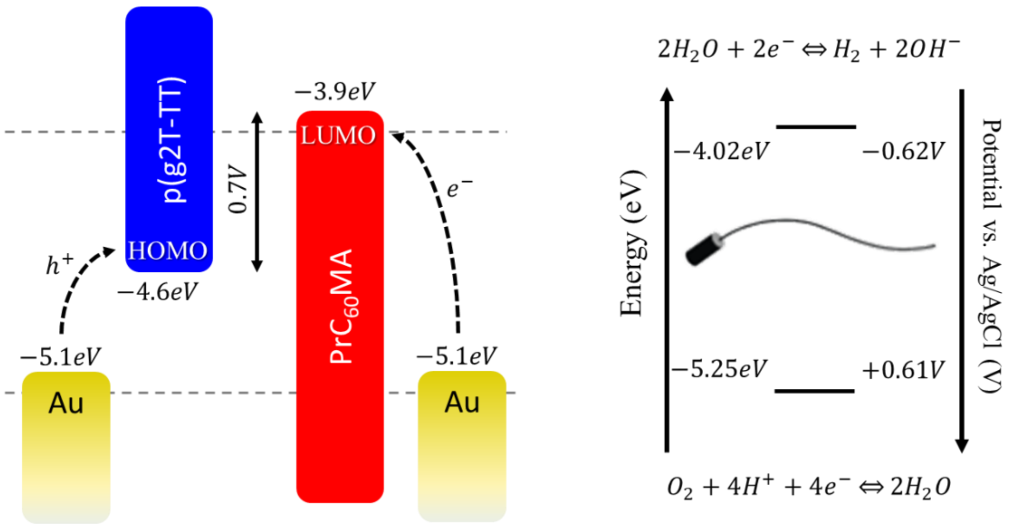

Supplementary Figure 6 | Energy band diagram and Electrochemical window. The energy levels of p(g2T-TT)[1] and $PrC_{60}MA$[2], work function of Au electrodes and the corresponding water electrochemical window at pH=7 vs Vacuum level and vs Ag/AgCl reference electrode[3]. The HOMO of p(g2T-TT) and the LUMO of $PrC_{60}MA$ are symmetric about ~-4.25eV, which corresponds to a voltage of +0.35V vs Ag/AgCl.

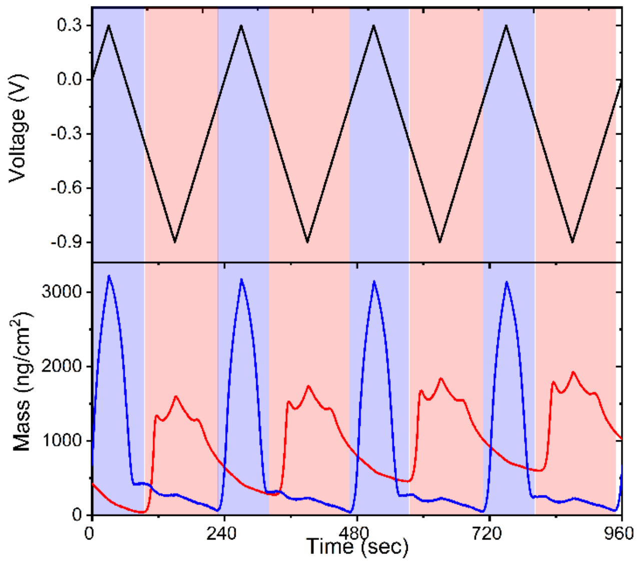

Supplementary Figure 7 | EQCM of pristine materials. Mass changes in EQCM measurement of p(g2T-TT) (blue curve) and $PrC_{60}MA$ (red curve), as calculated from the Sauerbrey equation for the 3rd overtone. Voltage applied between the gold-coated quartz sensor and an Ag/AgCl (3M KCl) reference electrode with a Pt wire CE. Scan rate was 10mV/sec.

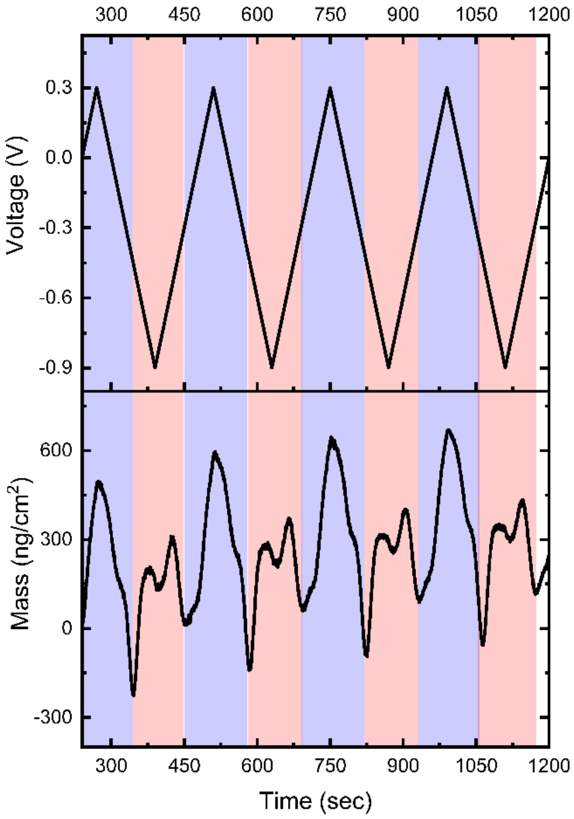

Supplementary Figure 8 | EQCM of 75:25 blend. Mass changes in EQCM measurement of a 75:25 w:w $PrC_{60}MA$:p(g2T-TT) blend, as calculated from the Sauerbrey equation for the 3rd overtone. Region marked in red are mass gain in the $PrC_{60}MA$ phase, blue regions are mass gain in the p(g2T-TT) phase. Voltage applied between the gold-coated quartz sensor and an Ag/AgCl (3M KCl) reference electrode with a Pt wire CE. Scan rate was 10mV/sec.

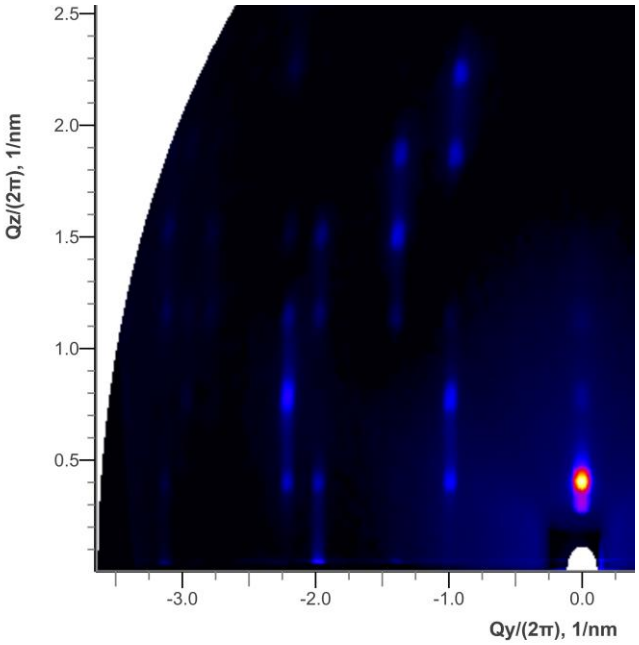

Supplementary Figure 9 | Grazing Incidence Wide-Angle Scattering (GIWAXS) of pristine $PrC_{60}MA$ annealed at 120°C for 20min.

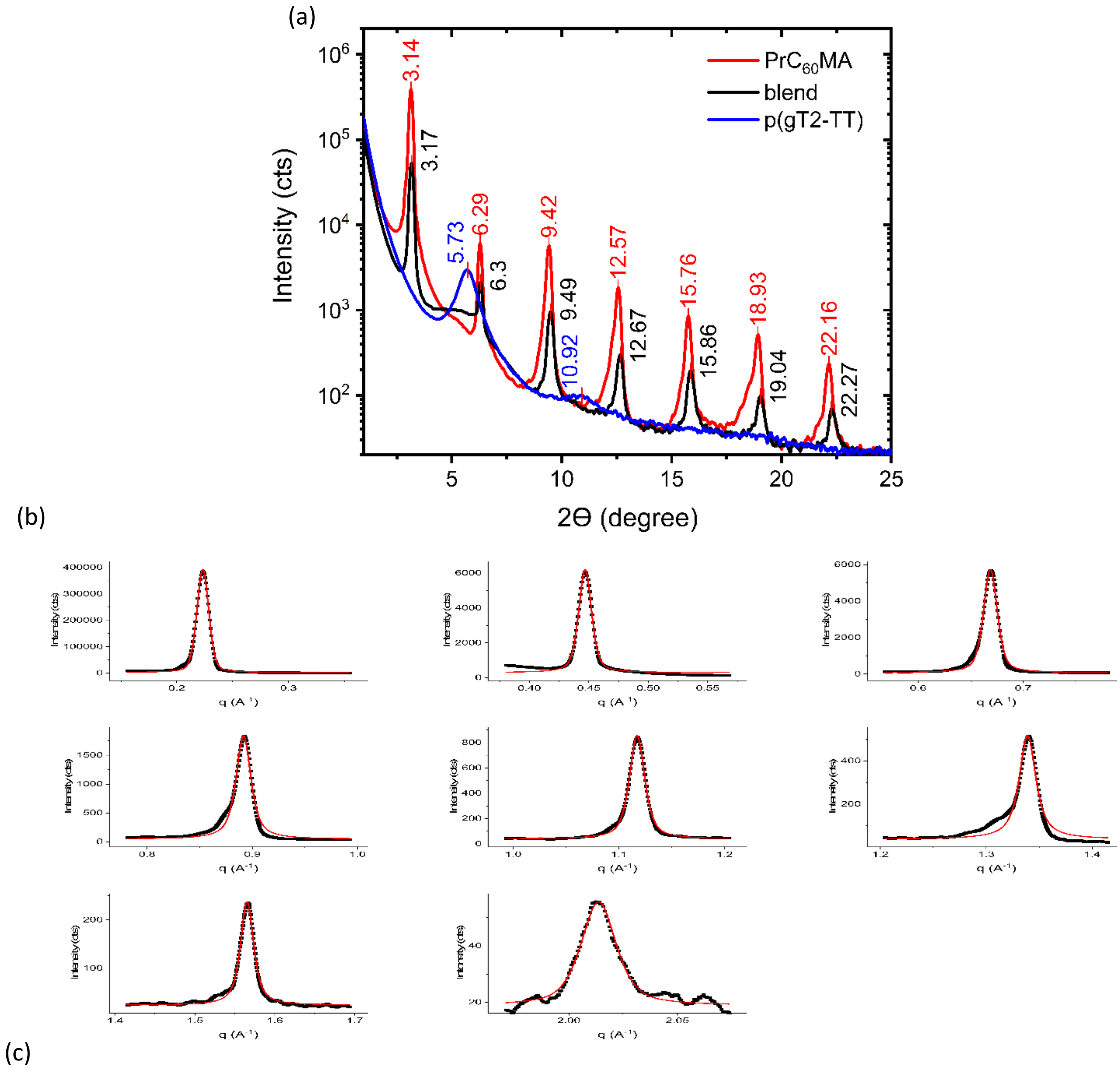

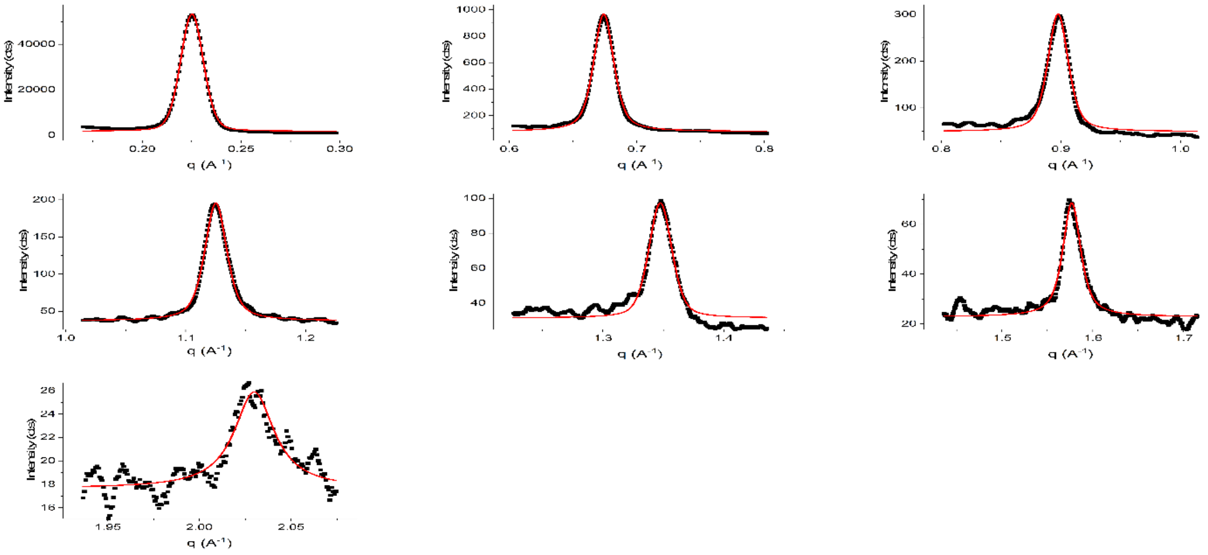

Supplementary Figure 10 | X-Ray Diffraction Peak Analysis of pristine materials and 95:5 blend. (a) Peak identification for the extraction of lamellar stacking d-space. (b) and (c) Peak FWHM fitting using Pseudo-Voigt function for peak orders 3-8 for the extraction of coherence length[4] for pristine $PrC_{60}MA$ and $PrC_{60}MA$ in the blend, respectively. First-order peaks and peaks that diverge significantly from the general trend are disregarded in the linear fitting

Supplementary References

1. Giovannitti, A. *et al.* Controlling the mode of operation of organic transistors through side-chain engineering. *Proc. Natl. Acad. Sci.* **113**, 12017–12022 (2016).

2. Li, C.-Z. *et al.* Effective interfacial layer to enhance efficiency of polymer solar cells via solution-processed fullerene-surfactants. *J. Mater. Chem.* **22**, 8574–8578 (2012).

3. Peljo, P. & Girault, H. H. Electrochemical potential window of battery electrolytes: the HOMO-LUMO misconception. *Energy Environ. Sci* **11**, 2306 (2018).

4. Rivnay, J., Noriega, R., Kline, R. J., Salleo, A. & Toney, M. F. Quantitative analysis of lattice disorder and crystallite size in organic semiconductor thin films. *Phys. Rev. B* **84**, 045203 (2011).
